# Stepwise partially overlapping primer-based PCR for genome walking

**DOI:** 10.1186/s13568-018-0610-7

**Published:** 2018-05-09

**Authors:** Kunpeng Chang, Qiong Wang, Xiaofei Shi, Shuixing Wang, Hongjing Wu, Lijuan Nie, Haixing Li

**Affiliations:** 1 State Key Laboratory of Food Science and Technology, Sino-German Joint Research Institute, Nanchang University, Nanchang, 330047 Jiangxi China; 2 College of Life Science and Engineering, Northwest Minzu University, Lanzhou, 730030 Gansu China

**Keywords:** Partially overlapping primer, PCR, Genome walking, *gadA* locus, *hyg*

## Abstract

A stepwise partially overlapping primer-based PCR (SWPOP-PCR) method for isolating flanking unknown DNA regions was developed, which comprises three rounds of nested PCRs sequentially driven by SWPOP primer-nested specific primer pairs. SWPOP primer set is characterized by a partial overlap of 10 bp with 3′-part of the latter primer is identical to 5′-part of the former one, which makes the SWPOP primer in use anneal to SWPOP site of the prior PCR product only at relatively low temperature. For each PCR, target single-stranded DNA primed by the SWPOP primer in the exclusive one low-stringency cycle is converted into double-stranded form in the following high-stringency cycle due to the presence of a perfect annealing site for the specific primer. This double-stranded DNA bounded by the specific primer and the SWPOP primer is exponentially amplified in the remaining high-stringency cycles. Non-target single-stranded DNA, however, cannot be amplified given the lack of perfect complementary sequences for any primers. Therefore, the partial overlap of a SWPOP primer set preferentially synthesizes target products but inhibits nonspecific amplification. We successfully exploited SWPOP-PCR to obtain the DNA sequences flanking glutamate decarboxylase gene (*gadA*) locus in *Lactobacillus brevis* NCL912 and hygromycin gene (*hyg*) integrated in rice.

## Introduction

Numerous PCR-based genome walking methodologies have been developed for identification and isolation of neighboring unknown DNA sequences adjacent to known genomic regions, which can be classified into three main categories (Kotik 2009; Leoni et al. 2011): (I) inverse PCR (Ochman et al. 1988); (II) ligation mediated PCR (Mueller and Wold 1989; Arnold and Hodgson 1991; Jones and Winistorfer 1992; Yan et al. 2003; Ji and Braam 2010); and (III) randomly primed PCR (Liu and Whittier 1995; Tan et al. 2005; Wang et al. 2013). The first two categories rely on labor-intensive and time-consuming restriction digestion and ligation of genomic DNA before PCR amplification (Rosenthal and Jones 1990; Acevedo et al. 2008; Leoni et al. 2008, 2010; Trinh et al. 2012b; Spalinskas et al. 2013). In addition, requirements of high-quality genomic DNA and several different restriction enzymes limit the actual utilization of these methods (Iwahana et al. 1994; Tsuchiya et al. 2009; Bae and Sohn 2010; Trinh et al. 2012a). The third category requires no complicated DNA manipulations before or after PCR (Liu and Chen 2007; Luo et al. 2011). However, the excessive accumulation of non-target DNA products as a consequence of nonspecific annealing of arbitrary primer is the major limitation of these methods (Terauchi and Kahl 2000; Reddy et al. 2008; Thirulogachandar et al. 2011).

Recently, we developed a partially overlapping primer-based PCR (POP-PCR) method for genome walking, which employed a set of POP primers having identical 3′ ends of 10 bp to suppress the amplification of non-target products while effectively enrich the target molecules. However, a separate POP primer has to be used in each round of nested POP-PCR, which contributes to the complexity of experimental operation thus prone to error (Li et al. 2015). Here, we present a novel genome walking strategy, termed stepwise partially overlapping primer-based PCR (SWPOP-PCR), which is easier to operate and more economical than the conventional POP-PCR. The feasibility of the new method was tested by retrieving segments of interest from the genomic DNA of *L. brevis* NCL912 and rice.

## Materials and methods

### Genomic DNA isolation and purification

The genomic DNA of *L. brevis* NCL912 (= CCTCCM208054) was isolated with the TIANamp Bacteria DNA Kit (Tiangen Biotech Co., Ltd, Beijing, China) according to the manufacturer’s instructions. Rice genomic DNA was kindly provided by Dr. Shaobo Li (Nanchang University).

### Oligonucleotide primers

The primers used in this study are summarized in Table 1. We designed four sets of SWPOP primer, and each set consists of three primers [SWPOP-P (primary PCR), SWPOP-S (secondary PCR), SWPOP-T (tertiary PCR)] in which 10 bp in 3′-part of the latter primer is identical to 10 bp of 5′-part of the former one. A gene-specific primer set consists of three nested primers [SP-P (primary PCR), SP-S (secondary PCR), SP-T (tertiary PCR)], which were designed based on the DNA sequences of glutamate decarboxylase gene (*gadA*) locus (GenBank accession number JX074764) of *L. brevis* NCL912 (Li et al. 2013) and hygromycin gene (*hyg*) (KF206149.1) integrated in the genome of rice, respectively. Each specific primer had a similar melting temperature (T_m_) with its paired SWPOP primer. Other rules in the design of primers were generally the same as those for normal PCR.

**Table 1:** Primers used in this study

Primer set	Stepwise partially overlapping primer set (SWPOP primer set)	Specific primer set (SP primer set)^a^	
		*gadA* locus	*hyg*
Primary PCR	CAGTCAGTCTCAGCTAGTCAGTGTC	TCCATACCCTCATCTCCATTTCCAT (− 1 to − 25)	CGGCAATTTCGATGATGCAGCTTGG (− 1 to − 25)
	CAGTCAGTCTCGTCAGTCGTGCAGT		
	CAGTCAGTCTCCACGTCACCAGTCA		
	CAGTCAGTCTAGCAGCAGTCGTCAG		
Secondary PCR		AACTATCACCCCACAACGTCATCTC (− 157 to − 181)	CGGGACTGTCGGGCGTACACAAATC (− 64 to − 88)
Tertiary PCR		ACCGTTCATAGGCGAAATTGTTTGT (− 335 to − 359)	GACCGATGGCTGTGTAGAAGTACTC (− 112 to − 136)

Four SWPOP-P primers were respectively paired with a specific primer for primary PCR; in the subsequent secondary or tertiary PCR, the SWPOP primer and a corresponding specific primer in the same row were matched. The partial identical sequences of SWPOP primers were underlined

^a^The bracketed numerical ranges below specific primers refer to coordinates in the known DNA sequences (a first base at the 5′-end of an outermost specific primer is defined as-1)

### PCR procedure

Three rounds of PCR (primary, secondary, tertiary) were carried out in each walking process using the previous PCR product as the template of the subsequent PCR. A specific primer was paired with its corresponding SWPOP primer(s) in each round of PCR. In the primary PCR reaction, the 50 μL reaction mixture contained 1 × LA PCR buffer II (Mg^2+^ plus), 400 μM of each dNTP, 0.2 μM of each primer, template DNA (10–100 ng for *L. brevis* NCL912 and 100–1000 ng for rice), and 2.5 U of TaKaRa LA Taq HS. In the secondary/tertiary PCR reaction, the 50 μL reaction mixture included 1 × LA PCR buffer II (Mg^2+^ plus), 400 μM of each dNTP, 0.2 μM of each primer, 1 μL of the previous round of PCR products, and 2.5 U of TaKaRa LA Taq HS.

Each round of PCR contained three annealing stages: stage 1, five high-stringency (65 °C) cycles; stage 2, one low-stringency (25 °C) cycle in primary PCR or one reduced-stringency (50 °C) cycle in secondary/tertiary PCR; and stage 3, twenty-five high-stringency (65 °C) cycles. Reaction profiles of the three rounds of PCR are presented in Table 2.

**Table 2:** Thermal cycling parameters used in SWPOP-PCR method

Round of PCR	Stage	Thermal condition	Cycle number
Primary		94 °C 2 min	1
	1	94 °C 30 s, 65 °C 1 min, 72 °C 2 min	5
	2	94 °C 30 s, 25 °C 1 min, 72 °C 2 min	1
	3	94 °C 30 s, 65 °C 1 min, 72 °C 2 min	25
		72 °C 5 min	1
1 μL of the product was directly used as plate in the secondary round of PCR			
Secondary		94 °C 2 min	1
	1	94 °C 30 s, 65 °C 1 min, 72 °C 2 min	5
	2	94 °C 30 s, 50 °C 1 min, 72 °C 2 min	1
	3	94 °C 30 s, 65 °C 1 min, 72 °C 2 min	25
		72 °C 5 min	1
1 μL of the product was directly used as plate in the tertiary round of PCR			
Tertiary	Reaction profile of tertiary PCR is identical to that of secondary PCR		

### DNA manipulation and sequencing

PCR products were purified with the MiniBEST Agarose Gel DNA Extraction Kit Ver.4.0 (TaKaRa, Dalian, China), and were directly sequenced by Sangon Biotech Co., Ltd. (Shanghai, China).

## Results

### Overview of stepwise partially overlapping primer-based PCR

**Fig. 1:**
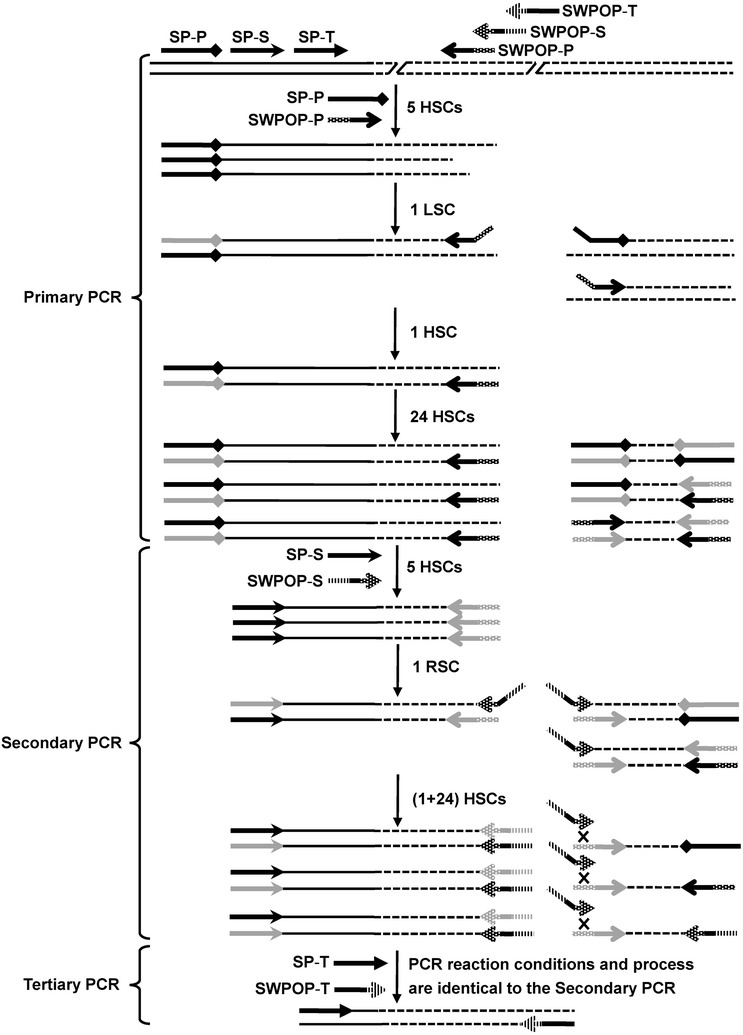
Outline of stepwise partially overlapping primer-based PCR. The first five high-stringency cycles (HSCs) of each PCR were carried out to increase the copy number of specific single-stranded DNA (ssDNA) of interest with different length. The one low-stringency cycle (LSC) of primary PCR allowed SWPOP-P to anneal to the target DNA and extend towards SP-P. The one reduced-stringency cycle (RSC) of secondary/tertiary PCR allowed SWPOP-S/SWPOP-T to bind to the prior SWPOP complementary site(s). A double-stranded target DNA molecule was obtained in the first HSC following LSC/RSC, and served as the template for the remaining twenty-four HSCs. Non-target amplification was suppressed because the double-stranded form could not be synthesized from a non-target single strand. Drawings on the right side: potential nonspecific (non-target) amplifications; SP-P, SP-S, and SP-T: specific primer for primary, secondary, and tertiary PCR, respectively; SWPOP-P, SWPOP-S, and SWPOP-T: stepwise partially overlapping primer for primary, secondary, and tertiary PCR, respectively; solid lines: the known sequence; dotted lines: the unknown sequence; grey arrows: primers complementary sites

The outline of SWPOP-PCR is illustrated in Fig. 1. SWPOP-PCR involves three successively nested PCRs. The key point of this strategy is to design a set of stepwise partially overlapping primers in which 3′-part (10 bp) of the latter SWPOP primer is identical to 5′-part of the former one, hence annealing between the SWPOP primer and its partially complementary site (prior SWPOP site) only occur at relatively low temperature. In the initial five high-stringency cycles (HSCs), the SP primer (specific primer) annealed to its complementary site within the known sequence and extended towards the unknown region, thus increased the copy number of single-stranded DNAs (ssDNAs) of interest with different length (Deng et al. 2010). The subsequent low-stringency cycle (LSC)/reduced-stringency cycle (RSC) allowed SWPOP primer (walking primer) to create annealing site(s) adapted for its 3′-end/bind to the former SWPOP site(s) and extend within the genomic DNA or the previously generated ssDNAs of interest, newly producing a pool of ssDNAs consisting of non-target and target fragments with SWPOP primer sequence at the 5′-ends, among which the target ssDNAs contained SP primer complementary sequence at the 3′-ends whereas the non-target ssDNAs had no perfect annealing sites for the both primers. A nascent target ssDNA was converted into double-stranded molecule bound by the both primers attributed to the presence of SP primer complementary sequence at the 3′-end in the following HSC, rather a non-target ssDNA could not be formed into double-strand ascribable to the absence of annealing sites for any primers. Therefore, the target molecule but not the non-target DNA was exponentially amplified equivalently to a specific PCR in the remaining HSCs.

### Genome walking of the *gadA* locus in *L. brevis* NCL912 and *hyg* integrated in rice

**Fig. 2:**
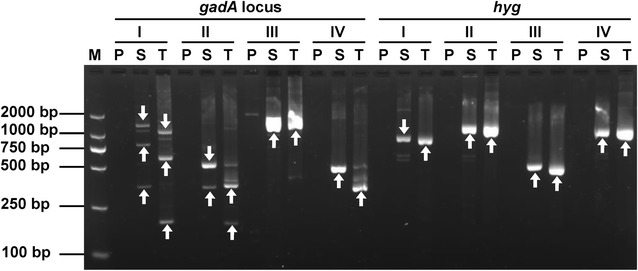
Genome walking of the *gadA* locus of *Lactobacillus brevis* NCL912 and *hyg* of rice. Each walking experiment contained four sets of PCR reactions that respectively utilized the four SWPOP primer sets, SWPOP1 (I), SWPOP2 (II), SWPOP3 (III) and SWPOP4 (IV), paired with a specific primer set. For each set of PCR reactions, the results of primary PCR (P), secondary PCR (S) and tertiary PCR (T) are presented. White arrows indicate target bands. M: DL2000 DNA marker

To confirm the validity of SWPOP-PCR, we employed the method to isolate target segments flanking the regions of *gadA* locus in *L. brevis* NCL912 and *hyg* integrated in rice. As shown in Fig. 2, four sets of parallel PCR reactions were successfully performed for each DNA sample, respectively. One or more clear predominant DNA band(s) appeared after the secondary and tertiary PCR reactions. A flanking region of approximate 1.2–1.6 kb in size was uncovered in each walking. Sequencing the bands in the tertiary PCRs showed that the obtained sequences were completely overlapped with the ends of the known gene region. In some cases, more than one target DNA bands suggested that a SWPOP-P primer annealed to multiple sites of a DNA plate.

We also sequenced the distinct DNA bands appeared in the secondary PCRs, and the results confirmed that all those distinct bands were target products. In most cases, a DNA band in secondary PCR was a little larger than that in the corresponding tertiary PCR in size due to the application of nested specific primers (Liu and Whittier 1995).

## Discussion

The key to the success of the proposed method is to have a possible annealing site in the unknown DNA sequence for SWPOP-P primer (SWPOP primer for primary PCR). In our PCR method, a SWPOP-P primer should find an adapted site in the plate given the fact that one super low-stringency (25 °C) cycle was performed in primary PCR. To increase the success rate in genomic walking, we designed four parallel SWPOP-P primers having heterologous 15 bp at the 3′-parts and identical 10 bp at the 5′-parts. The four heterologous 3′-parts are expected to guarantee at least one SWPOP-P primer creates an annealing site adapted for this SWPOP-P within genomic DNA (Parker et al. 1991; Parks et al. 1991).

In the traditional POP-PCR, three POP primers of each POP primer set have uniquely homologous 3′-parts and completely heterologous 5′-parts, thus four POP primer sets include (4 × 3) twelve primers (Li et al. 2015). In SWPOP-PCR, given that the 10 bp 5′-parts of the four SWPOP-P primers are identical, we designed only one SWPOP-S primer (for secondary PCR) with 10 bp of its 3′-part identical to the 5′-ends of all the four SWPOP-P primers, and automatically only one SWPOP-T primer (for tertiary PCR) with 10 bp of its 3′-part identical to the 5′-part of SWPOP-S primer. Therefore, four SWPOP primer sets contain only (4 + 1 + 1) six primers. Moreover, we could make mix master for secondary/tertiary SWPOP-PCRs due to the reaction reagents are the same except for the DNA plate. In addition, two rounds of PCR are sufficient to obtain satisfactory results, so the tertiary SWPOP-PCR can be omitted generally.

In genome walking methods using unspecific (walking) primers, three types of non-target products could be produced: (I) those primed by specific primer alone, (II) those primed by both specific primer and walking primer, and (III) those primed by walking primer alone (Arnold and Hodgson 1991; Bae and Sohn 2010; Wang et al. 2011). Types I and II non-target products could be easily excluded in the following nested PCR given the fact that an inner nested specific primer was used (Yan et al. 2003; Tan et al. 2005; Wang et al. 2007). The common problem faced by all these PCR techniques is how to get rid of type III nonspecific products (Liu and Whittier 1995; Deng et al. 2010; Thirulogachandar et al. 2011), which has limited the application of the existing genome walking methods. We herein use the arbitrary primer (walking primer or SWPOP primer) partially overlapping strategy to remove this type of undesired products. The partially overlapping characteristic made the SWPOP primer in use anneal to the former SWPOP primer complementary site of type III product only once because the exclusive one LSC/RSC was performed in the PCR, and primed the synthesis of a novel non-target ssDNA with the 3′-end still completely complementary to the former SWPOP primer, which lacked perfect annealing sites for any primers used in the current round of PCR. This new ssDNA could not be converted into double-stranded form in the following HSCs and thus could not be efficiently amplified. Clearly, type III non-target products were also readily diluted out by the SWPOP primer used in the next round of PCR. In theory, the SWPOP-PCR method is equivalent to a regular specific PCR in the amplification of the target fragments and the inhibition of non-target fragments.

In conclusion, we have developed a novel SWPOP-PCR method for genome walking to isolate and identify the unknown DNA sequences flanking the known segments. Compared to the conventional POP-PCR, SWPOP-PCR has the merits of simplicity and efficacy due to requiring fewer primers and being suitable for making mix master. The SWPOP-PCR is an alternative of the existing genome walking methods.

## Abbreviations

SWPOP-PCR: stepwise partially overlapping primer-based PCR
POP-PCR: partially overlapping primer-based PCR
*gadA*: glutamate decarboxylase gene
*hyg*: hygromycin gene
*L. brevis* NCL912: *Lactobacillus brevis* NCL912
SWPOP-P, SWPOP-S, and SWPOP-T: stepwise partially overlapping primer for primary, secondary, and tertiary PCR, respectively
SP-P, SP-S, and SP-T: specific primer for primary, secondary, and tertiary PCR, respectively
T_m_: melting temperature
HSC: high-stringency cycle
LSC: low-stringency cycle
RSC: reduced-stringency cycle
ssDNA: single-stranded DNA
